# The impact of a healthy checkout intervention on fruit and vegetable ‘micro-pack’ purchases in New Mexico

**DOI:** 10.1017/S1368980022002026

**Published:** 2022-12

**Authors:** Stephanie Rogus, Joanne Guthrie, Mihai Niculescu, Lina Xu

**Affiliations:** 1 Department of Family and Consumer Sciences, New Mexico State University, MSC 3470, P.O. Box 30003, Las Cruces, NM 88003, USA; 2 Economic Research Service, U.S. Department of Agriculture, Washington, DC, USA; 3 Marketing Department, New Mexico State University, Las Cruces, NM, USA; 4 Marketing Department, The Pennsylvania State University, Abington, PA, USA

**Keywords:** Healthy checkout, Grocery store, Produce, End-caps, Purchase behaviour, New Mexico

## Abstract

**Objective:**

Produce sold as plastic-wrapped packs of two to four individual items (i.e., produce micro-packs) that are low cost and placed at checkout may appeal to shoppers with budget constraints and provide a second chance to purchase items available elsewhere in the store. This study examined the impact of an intervention that placed produce micro-packs at checkout and promoted them in grocery stores across New Mexico, USA.

**Design:**

This quasi-experimental study placed produce micro-packs at checkout end-caps in thirteen stores (group 1), with eight stores serving as controls (group 2) from 1 July 2019 through 31 January 2020 (first phase). The intervention was extended to group 2 stores from 1 February 2020 through 30 June 2020 (second phase). Cashiers were directed to upsell the micro-packs to Special Supplemental Nutrition Program for Women, Infants, and Children recipients who had unspent cash value benefits for produce purchases.

**Setting:**

Twenty-one grocery stores across New Mexico.

**Participants:**

Twenty-one produce items sold as micro-packs in stores from July 2019 through June 2020.

**Results:**

A random effects model showed that the daily sales of micro-packs increased by 47 % during each intervention period. Group 2 stores had lower sales than group 1 stores during the first phase of the intervention. Once extended to group 2 stores, sales of micro-packs in those stores increased and sales in group 1 stores continued at the higher level.

**Conclusions:**

Placing produce micro-packs at checkout may increase produce sales and support health promotion efforts by public and private stakeholders.

Dietary patterns that follow the Dietary Guidelines for Americans are associated with lower risk of chronic disease^(1,2)^. The Dietary Guidelines for Americans recommend consumption of nutrient-dense foods and beverages and a dietary pattern consisting of wholegrains, lean meats and vegetable protein, low-fat dairy products and fruits and vegetables^(1)^. However, American diets continue to fall short of Dietary Guidelines for Americans recommendations, particularly for fruit and vegetable consumption^(3,4)^. While most Americans fail to consume recommended amounts of fruits and vegetables, diets of low-income Americans participating in federal food assistance programmes are of particular concern. The Supplemental Nutrition Assistance Program (SNAP) provides low-income Americans with funds that can be used to purchase foods in most grocery stores; in 2020, 39·9 million people participated in SNAP each month^(5)^. Although benefits are based on a Thrifty Food Plan (TFP) that is designed to provide enough funds to meet dietary recommendations, participants buy fewer servings of fruits and vegetables than recommended and report purchasing fewer fruits and vegetables compared with low-income and higher-income non-participating Americans^(3)^. The TFP assumes SNAP households should spend 40 % of their benefits on fruit and vegetables^(6),^^1^ but a 2016 Food and Nutrition Service study of purchases indicated they actually spend less than 15 % of their benefits on these foods^(8,9)^. SNAP is the largest food assistance programme serving low-income Americans; however, a second programme, the Special Supplemental Nutrition Program for Women, Infants, and Children (WIC), also provides food benefits to low-income pregnant, postpartum and breastfeeding women as well as infants and children under 5 years of age. This programme served 6·2 million people in an average month in 2020^(5)^. Unlike SNAP, which provides funds that can be used to purchase almost all foods sold in supermarkets and grocery stores, WIC funds the purchase of specific foods chosen to meet the needs of its target population, for example, milk, eggs, iron-rich cereals and whole grains.

Updates to food assistance programmes have been made in recent years to encourage the consumption of fruits and vegetables by recipients. In 2009, WIC added cash value benefits (CVB) to be used specifically for the purchase of fruits and vegetables, and the United States Department of Agriculture (USDA) has funded a number of projects that provide incentives to SNAP participants for purchasing fruits and vegetables^(10–12)^. In 2021, after the previously cited studies of fruit and vegetable purchasing by SNAP participants were conducted, the TFP was revised, with the result that benefit levels were increased^(7,13)^. The new TFP assumes 38 % of benefits should be allocated to fruits and vegetables. Given that the change in benefit level has just occurred, there is no information on whether SNAP shoppers will respond to the higher benefit levels by purchasing more fruits and vegetables. Simulations of likely purchasing changes in response to increased benefits suggest SNAP households may purchase more fruits and vegetables, but the estimated changes would not be large enough to assure that most households would meet recommendations^(14,15)^. Other preferences, such as a desire for convenience, may compete for use of the food dollar^(16)^, so strategies to encourage fruit and vegetable purchasing and make it more salient to consumers may still be valuable. Both WIC and SNAP include nutrition education components that promote fruit and vegetable consumption.

Despite these efforts, fruit and vegetable purchasing by low-income consumers continues to lag, and some efforts do not seem to be achieving their full potential. For example, research examining WIC CVB redemption in several states has found that recipients redeem about 70 % of the benefits and an evaluation of the Food Insecurity Nutrition Incentive Program (now called the Gus Schumacher Nutrition Incentive Program or GusNIP), a grant-funded programme operated by the USDA that is designed to incentivise purchase of healthy foods such as fruits and vegetables, found that recipients redeem 82 % of their benefits^(11,17–19)^. Point of purchase interventions has been proposed as a mechanism to increase overall produce purchases by food assistance recipients and the general public^(20,21)^.

Supermarket interventions have the potential to increase purchases of fruits and vegetables as the majority of household food is acquired from these outlets in the USA^(21–23)^. Consumers report that product variety and packaging, price, promotion and display location influence their purchasing decisions, and research suggests that manipulating these aspects of the in-store marketing environment can encourage healthier food purchases^(20,24)^. Behavioural economics theory suggests that consumers are not always rational decision-makers; psychological influences play a role in food choice, which can lead consumers to value short-term preferences, like taste, and to choose products with high visibility or attractiveness when they are feeling tired, rushed, distracted or hungry^(26)^. The behavioural economics concept of cognitive overload is experienced in stores due to the sheer number of food products available, time constraints of shoppers and distractions like shopping with children^(21)^. Healthier foods placed at checkout aisle end-caps can address time constraints by signalling convenience and address attention constraints by signalling prominence^(21)^. All customers must pass through checkout, and often wait in line, so low-cost, healthy items displayed at checkout may increase their attractiveness and encourage shoppers to purchase them^(21)^. For low-income shoppers in particular, items such as fruit and vegetable micro-packs, or plastic-wrapped packages of one or more fresh fruits or vegetables displayed on a rack at the checkout aisle, priced at around $1 may be appealing.

Studies examining the stocking policies of supermarkets have found that stores with consistent policies about replacing unhealthy items with healthier items at checkout were associated with fewer purchases of unhealthy items^(27,28)^; however, few studies have tested healthy checkout interventions in real-world settings. In these healthy checkout studies, researchers replaced less healthy items with healthier items, added healthier items to the current selection or removed less healthy items without replacing them with healthier items at one or more checkout aisles in a store. Interventions have ranged in duration from 4 d to 6 months, substituting unhealthy items like candy and soda with fresh fruits and vegetables, dried fruit, cereal bars, nuts, dried fish and bottled water or removing unhealthy items altogether. Five studies added healthy items to checkout aisles and found that sales of healthier items increased^(21,29–31)^, but there was no reduction in the sales of less healthy items^(29–31)^. Three studies substituted unhealthy foods with healthier options at checkout aisles and reported mixed results. Sigurdsson *et al.* found an increase in healthy food sales and decrease in unhealthy food sales^(33)^, whereas Huitink *et al.* found that participants purchased fewer of the healthy items at checkout, suggesting that they did not substitute less healthy items with healthier ones^(31)^. Adjoian *et al*. reported that a higher percentage of customers using the healthy checkout purchased healthy items compared with customers using the standard checkout; however, only 4 % of customers bought anything at checkout, so the impact of the healthy checkout aisle was likely limited^(34)^. Vogel *et al.* reported increased purchases of fruits and vegetables when unhealthy foods were removed from the checkout aisle and produce was placed near the entrance of stores^(35)^.

Only seven healthy checkout interventions have included fresh produce, and of those, five were experimental or quasi-experimental studies that included control stores or checkout aisles. Two of the five placed healthy products on a rack that was added to the checkout aisle^(21,30)^, two replaced the entire product selection at checkout with healthy items for one or more aisles^(29,34)^ and one only removed less healthy products from checkout aisles^(37)^. Three of the five studies reported increased sales of fresh fruits and/or vegetables^(21,29,35)^, whereas the other two could not report on changes in produce sales. One did not collect data on pre-intervention sales of fruits and vegetables and could not conclude anything about changes in produce purchases^(30)^ and one only examined changes in the purchase of healthy items overall, which included fresh and dried produce, granola bars, nuts, bottled water and other healthy items^(34)^. This study reported increased purchases of healthy items among shoppers who went through the healthy checkout aisle, noting that fresh and packaged fruit were the most purchased healthy product^(34)^. Of the three studies that reported increased fresh produce purchases, one reported an increase in the purchase of carrot snack packs (out of five total healthier items) but no increase in fresh fruit purchases^(29)^, the second found that fresh produce micro-pack sales increased while overall sales stayed constant, suggesting that the micro-packs increased fruit and vegetable purchases^(21)^ and the third reported improvements in dietary quality among their female participants in addition to storewide decreases in unhealthy food sales and increases in fruit and vegetable sales^(35)^. Payne *et al.* also examined sales of produce micro-packs purchased using SNAP, finding increased purchases of micro-packs and an increase in the micro-packs’ share of SNAP spending^(21)^. They concluded that fruit and vegetable micro-packs can replace purchases of other foods for SNAP recipients^(21)^. Although low-income shoppers may not purchase certain snacks at checkout due to their relative expense compared with multi-pack snacks throughout the store^(30)^, these studies suggest that offering low-cost produce micro-packs at checkout may be a promising strategy for increasing produce purchases of low-income shoppers and encouraging full redemption of benefits specifically targeting fruit and vegetable purchasing, such as the WIC CVB, though more studies testing such interventions are needed.

More research is needed to determine the effectiveness of various in-store strategies, including healthy checkouts, particularly for low-income consumers^(20,36)^. Of the food retail interventions conducted, most did not include control or comparison groups, were not experimental and included subjective outcome measure data such as self-reported purchases^(36)^. Most studies also did not examine the sustained effects of the intervention over time; less than 20 % of studies analysed intervention effects beyond 3 months^(36)^.

This study tests a healthy checkout intervention whereby low-cost fresh fruit and vegetable micro-packs were sold and promoted at checkout aisle end-caps. The purpose of this study is to examine the impact of a healthy checkout intervention on fruit and vegetable micro-pack purchases across stores of a regional grocery chain in New Mexico that serves a low-income customer base. This research extends previous research by testing the intervention over a longer time period, including an objective outcome measure and including more intervention and control stores.

## Methods

### Study design

This research was part of a larger intervention aimed at increasing the redemption of WIC CVB. In partnership with a regional grocery store chain, the intervention included placing fruit and vegetable micro-packs on racks at checkout aisles and changing the software in store registers to notify cashiers when WIC recipients had additional money left on their CVB. Before the intervention began, cashiers were trained to provide information to recipients on the amount left on their CVB and to upsell the micro-packs. However, subsequent training was not provided to any new cashiers that may have been hired, and consistency of cashier upselling was not monitored during the intervention. Therefore, the frequency of cashier upselling may have declined over time.

Fruit and vegetable micro-packs were sold as plastic-wrapped packs of two to four individual fresh fruits and vegetables. Micro-packs were already being sold in the produce aisle of each store prior to the intervention, and available micro-packs were taken from that aisle for the intervention. Micro-packs continued to be sold in the produce section during the intervention. The micro-packs were added to checkout aisles and did not displace the other products typically placed at checkout. Twenty-one different fruits and vegetables were sold as micro-packs for $0·20 to $2·79 each (Table 1).

**Table 1:** Fruit and vegetable micro-pack type, pack size and retail price

Fruit/vegetable	Pack size	Retail price
Roma tomatoes	3	$0·99
Anaheim pepper	2	$0·99
Red delicious apple	2	$0·99
Avocado	2	$2·79
Green bell pepper	2	$0·99
Grapefruit	2	$0·99
Green beans	2	$0·99
Jalapeno	4	$0·99
Lemon	3	$0·99
Lime	3	$0·99
Nectarine	3	$2·39
White onion	2	$0·99
Yellow onion	2	$0·99
Orange	2	$0·99
Peach	3	$2·39
Plum	4	$2·39
Potato	2	$0·99
Mexican squash	2	$0·99
Yellow squash	2	$0·99
Sweet potato	2	$0·99
Banana	1	$0·20

The aim of the intervention was to nudge consumers to purchase more fruits and vegetables by: (1) encouraging income-constrained consumers who may limit their fruit and vegetable purchasing while shopping over fear of overspending to purchase produce if they have money leftover at checkout and (2) encouraging impulse or unplanned purchases of produce by increasing their visibility at checkout^(37)^.

The intervention began on 1 July 2019 in thirteen stores (group 1) in New Mexico, with eight stores serving as controls (group 2). Group 1 and group 2 stores were selected in consultation with the retailer and are located in areas throughout the state, with group 1 stores located in the north, south, east, central and northwestern part of the state and group 2 stores located in the north, south, central and east. Additionally, the majority of stores participating in the intervention serve a low-income clientele; over 75 % of intervention and 60 % of control stores are located in census tracts categorised as ‘low-income’ by the United States Department of Agriculture’s Economic Research Service (USDA-ERS) (Table 2).

**Table 2:** Low-income population and location of group 1 and group 2 stores*

	Census tract low-income population (%)	Low-income census tract†	Location
Group 1 stores			
1	36	Yes	North, central
2	34	No	Southeast
3	62	Yes	East, central
4	0	No	North, central
5	76	Yes	Northwest
6	60	Yes	Northwest
7	52	Yes	North, central
8	41	Yes	South, central
9	69	Yes	South, central
10	34	No	South, central
11	49	Yes	North, central
12	68	Yes	East, central
13	33	Yes	Central
Group 2 stores			
1	0	No	North, central
2	32	No	Southeast
3	38	No	Northeast
4	57	Yes	South, central
5	59	Yes	Central
6	39	Yes	North, central
7	42	Yes	Northeast
8	53	Yes	Central

* Table created using the USDA-ERS food access research atlas.

† A low-income census tract is defined by the ERS as ‘tracts with a poverty rate of 20 % or higher, or tracts with a median family income less than 80 % of median family income for the state or metropolitan area’^(38)^.

The first phase of the intervention ran for 7 months in group 1 stores (through 31 January 2020). At that time, the second phase of the intervention was initiated where the intervention was extended to the eight group 2 stores (through 30 June 2020), while continuing in group 1 stores. Daily sales, in dollars, of each micro-pack by store were obtained from the retailer from 1 March 2019 through 30 June 2020.

### Statistical analysis

A random effects model was estimated to examine the change in average daily sales per store of the micro-packs in group 1 and group 2 stores during the intervention periods. The model included an indicator variable for store type (group 1 or group 2) and intervention time period (pre-intervention, intervention phase 1 and intervention phase 2), and an interaction between the two. The marginal means were then estimated for group 1 and group 2 stores during each time period.

The analysis was conducted using R version 4.0.2, and differences were determined to be statistically significant if the *P*-value was below 0·05^(39)^.

## Results

**Fig. 1:**
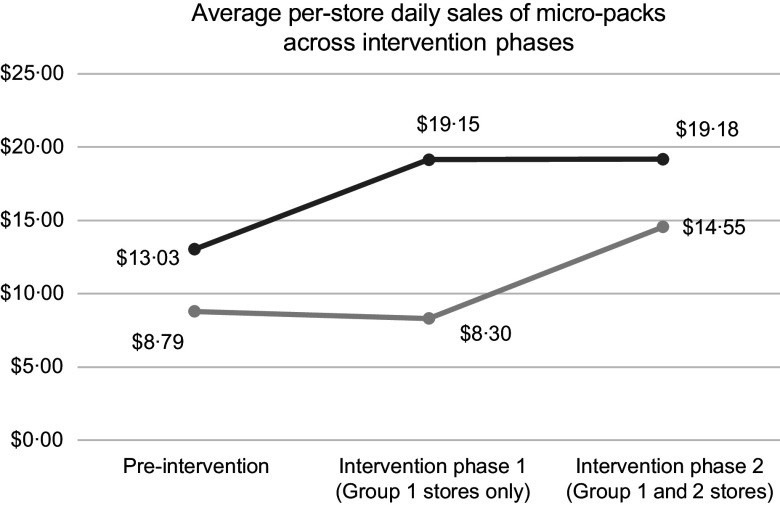
Average per-store daily sales of micro-packs for group 1 and group 2 stores across intervention phases. 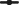, Group 1; 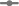, Group 2

In order to focus the analysis on stores in low-income areas, the two stores with 0 % low-income population were excluded from the analysis. Figure 1 shows the daily sales of all micro-packs at group 1 and group 2 stores, averaged across intervention phases. Sales in group 1 stores increased from baseline following both intervention periods. Sales in group 2 stores did not increase following the first phase of the intervention but did increase following the second phase, when they also participated in the intervention.

The results of the random effects model showed that sales increased during both phases of the intervention.^2^ Daily micro-pack sales (per store) in group 1 stores significantly increased by 47 % (*P* < 0·0001) from baseline (i.e. pre-intervention levels of micro-pack sales in group 1) during the first phase of the intervention, whereas sales in group 2 stores decreased by 3·76 % from baseline, though this change was insignificant (Table 3). During the second phase of the intervention when it was extended to group 2 stores, daily micro-pack sales (per store) in group 1 and group 2 stores increased significantly by 47·2 % (*P* < 0·0001) and 44·21 % (*P* < 0·0001) from baseline, respectively. The differences-in-differences change in average sales in group 1 stores during the first phase of the intervention was significantly higher compared with group 2 stores (+50·76 %, *P* < 0·0001). For group 2 stores, the change in average sales (+2·99 %) during the second phase of the intervention (*v*. baseline) was not significantly higher compared with group 1 stores. Taken together, these results demonstrate that sales increased in all stores after the intervention was implemented in those stores.

Calculating the marginal mean sales of group 1 and group 2 stores from the random effects model showed that group 1 stores had significantly higher sales of micro-packs during the first ($19·15, 95 % CI14·85, 23·4) and second ($19·18, 95 % CI 14·88, 23·5) phases of the intervention compared with the pre-intervention period in those stores ($13·03, 95 % CI8·71, 17·3) (Table 4). Group 2 stores did not have significantly higher sales during the first phase of the intervention ($8·30, 95 % CI 2·61, 14·0) compared with the pre-intervention period in those stores ($8·79, 95 % CI 3·09, 14·5) but did have higher sales during the second phase of the intervention ($14·55, 95 % CI 8·86, 20·2) compared with both the pre-intervention and first phase of the intervention (see Table 4).

**Table 3:** Percentage sales relative to pre-intervention in group 1 and group 2 stores (baseline = 100 %)*

Dependent variable	Group	Pre-intervention (% of baseline for group 1 stores)	Phase 1 intervention (% of baseline for group 1 stores)	Difference %	*P*	Difference-in-differences %	*P*
Daily micro-pack sales per store	Group 1	100·00	147·00	47·00	*P *< 0·0001	50·76	*P* < 0·0001
	Group 2	67·46	63·70	−3·76	*P* = 0·26		
Dependent variable	Group	Pre-intervention (% of baseline for group 1 stores)	Phase 2 intervention (% of baseline for group 1 stores)	Difference		Difference-in-differences	
Daily micro-pack sales per store	Group 1	100·00	147·20	47·20	*P* < 0·0001	2·99	*P* = 0·51
	Group 2	67·46	111·67	44·21	*P* < 0·0001		

* The statistical analyses are based on sales (not percentages) and percentages shown in tables were calculated relative to the pre-intervention sales in group 1 stores (baseline).

**Table 4:** Average daily micro-pack sales per store by intervention phase, in USD

Variable	Estimate	95 % CI
Group 1		
Pre-intervention (no intervention)	$13·03	8·71, 17·3
Intervention phase 1 (active intervention group)	$19·15	14·85, 23·4
Intervention phase 2 (active intervention group)	$19·18	14·88, 23·5
Group 2		
Pre-intervention (no intervention)	$8·79	3·09, 14·5
Intervention phase 1 (no intervention)	$8·30	2·61, 14·0
Intervention phase 2 (active intervention group)	$14·55	8·86, 20·2

## Discussion

Results of this study showed that the total daily sales of fruit and vegetable micro-packs increased following the first phase of the intervention in group 1 stores. Sales also increased in group 2 stores during the second phase of the intervention when they no longer acted as a control group and participated in the intervention. The increase in micro-pack sales in group 1 stores remained significantly higher than the pre-intervention period during the second phase of the intervention, demonstrating a sustained effect of the intervention in those stores. These results are similar to other studies that have examined the impact of healthy checkout interventions, which found that healthy purchases increased during the intervention period^(21,29–35)^.

Few studies have tested healthy checkout interventions that include fresh produce. Of those that included a control group, all were conducted over a relatively short timeframe – from 2 weeks to 6 months^(21,29,30,34,35)^. Three reported increases in produce purchases, with one finding vegetables^(29)^, one finding fruits and vegetables^(37)^ and one finding fruit and vegetable micro-packs increased in sales^(21)^. The latter study is the only study that has exclusively tested a produce micro-pack intervention. It was conducted over 1 month in three stores (one control and two intervention stores) and reported between 80 and 300 % increase in sales of micro-packs in the intervention stores compared with control stores during the intervention period^(21)^. Our results align with this study, and the longer period of time over which this study was conducted allowed for the examination of the 7-month intervention and longer-term effects of the intervention through 1 year.

Although the increase in average per-store daily sales in intervention and control stores during the intervention – ranging from $5·76 to $6·15 – may seem small, other studies, including those that offer cash incentives, show relatively small increases in fruit and vegetable purchasing^(40)^. However, these small changes may be meaningful: a recent study examining the increase in spending needed by SNAP recipients in order to satisfy dietary recommendations for fruit and vegetable consumption found that recipients do not need to spend a lot more money in order to meet recommendations while increasing their produce variety^(8)^. Using simulation for a four-person household that is receiving the maximum monthly SNAP benefits, they show that produce recommendations cannot be met by spending 25 % of food dollars on fruits and vegetables. In order to meet recommendations and increase variety, recipients need to increase their expenditures to 30 % and ideally 40 %. This means spending $8·37 and $24·12 more per household, respectively, than Americans are currently spending on produce^(8)^. Further, SNAP households tend to overspend in categories such as fat, oils and sweets, which can be reduced and money can be shifted to fruits and vegetables^(9,21)^.

With the increasing awareness of the relationship between diet-related disease, low income and food insecurity, policy interest in nutrition security has grown. Nutrition security refers to a focus on consistent access to affordable healthy foods and beverages that may help prevent and treat disease^(41)^. Aligning with this focus is increasing interest in promoting purchases of produce in particular by lowering a household’s cost of purchasing produce through expansion of existing programmes like the WIC CVB and supporting new programmes such as GusNIP, bonus bucks and produce prescription programmes^(42,43)^. The recent update to the TFP will permanently increase SNAP benefits, increasing the purchasing power of recipients and potentially supporting additional fruit and vegetable purchases^(7)^. However, for these programmes to succeed in promoting healthy diets, purchasing behaviour must also change^(44,45)^. An evaluation of the Healthy Incentive Pilot that provided financial bonuses to SNAP participants for fruit and vegetable purchasing found evidence suggesting that informational or promotional aspects of the programme were important contributors to success.^(40)^

USDA supports nutrition education targeted to SNAP participants and other low-income individuals through its SNAP-Ed programme^(46)^. SNAP-Ed is encouraged to work in a variety of community settings, including supermarkets and grocery stores that serve large numbers of low-income consumers^(46)^. These findings may be of interest to SNAP-Ed and other nutrition education programmes that include a focus on encouraging purchase of fruits and vegetables. Nutrition promotion programmes focused on supermarket interventions have been investigated in a wide range of nations, including Australia, the United Kingdom (UK), Norway, Canada, Japan and the Netherlands^(47)^; therefore, replication in other settings could be of interest. In fact, the UK recently announced restrictions on the placement of foods high in sugar, fat and salt at checkouts and other prominent locations in medium and large food retailers, including supermarkets^(48)^. The potential success of this policy was demonstrated by a study that removed chocolate confectionery from prominent locations in stores, including the store entrance and aisle end-caps. The study found that the seasonal increase in confectionery sales was attenuated in intervention stores compared with control stores, which resulted in significant reductions in total energy and fat purchases^(49)^.

Nudges are often encouraged as a low-cost strategy, but it is important to consider the feasibility and sustainability of these approaches. In the case of this project, retailers assumed the cost of packaging and placement of items, making it potentially feasible to implement more widely, assuming continued retailer interest and support. This intervention added healthy items to the checkout aisle without removing any less-healthy alternatives, which may have affected the impact of the intervention but may also be a more sustainable alternative for retailers. Fruits and vegetables placed at checkout may compete with other products that incur slotting fees, making retailers possibly less willing to modify their current checkout selection. However, some food assistance benefits and incentives, such as WIC CVB and fruit and vegetable purchasing incentives or ‘bonus bucks’ offered through some programmes^(12)^, can only be used to purchase produce. This restriction may incentivise retailers to consider adding produce to the checkout aisle, particularly because these products have high profit margins compared with other product categories^(50)^. As of June 2022, almost 3 years after these data were collected, the stores that implemented this intervention were still offering the micro-packs at checkout, indicating the potential for long-term acceptance. This type of intervention could appeal to retailers to nudge purchasing of fruits and vegetables and as a demonstration of corporate social responsibility.

Healthy checkout interventions may be particularly effective because of their impact on impulse purchases. The convenience and prominence of items at checkout are attractive to customers, and offering low-cost, healthier options provides healthier alternatives for impulse purchases and signals a discount to shoppers^(21)^. For shoppers using food assistance benefits, these items offer a second chance to fully redeem their benefits.

The strengths of this study are that it was quasi-experimental and included control stores, it included an objective outcome measure and it was conducted over 12 months, which enabled the sustained effects of the intervention to be captured. To our knowledge, published studies have not examined the impact of a healthy checkout intervention beyond 6 months and this study provides evidence of sustained effects of these interventions. This study also included produce that is low cost for consumers or most commonly purchased^(8,9)^; twelve out of twenty-one of the fruits and vegetables fell into either of these categories. The limitations of this study are that consumption was not captured and data were not available on the form of payment for purchases of the micro-packs – WIC, SNAP or cash – making it impossible to assess to what extent it affected participant use of programme benefits. Second, because this was an intervention that combined placement of low-priced micro-packs at the checkout aisle and promotion of them (through cashier upselling), it is unclear whether micro-pack placement, price or promotion had a greater impact on purchases. Third, the promotion of the micro-packs may not have been sustained over the study period due to no follow-up training or monitoring to ensure that cashiers continued to promote the micro-packs to WIC recipients. Although the regression results showed that, overall, there was a sustained increase in micro-pack sales in intervention stores during the second intervention period, it is worth noting that stores varied in their sales of micro-packs over the study period, with some stores showing a drop in sales a few months after each intervention period. Further research may help identify strategies to maintain interest over time, perhaps by incorporating occasional additional promotional strategies to refresh the message. Fourth, data were only provided on micro-pack sales, not overall produce sales, so it is unclear whether the micro-packs increased fruit and vegetable purchases as customers may have shifted their current produce purchases to the micro-packs without buying more produce overall. Future research is needed to further assess sustained impacts of various types of healthy checkout interventions on the purchase and consumption of produce. Future research is also needed to differentiate the impact of various intervention strategies – such as product, placement, promotion and price – on produce purchases.

## Footnotes

1 The 40 % figure is based on the 2006 TFP. It was the most recent update until 2021 and included adjustments for inflation but kept the real value of the TFP unchanged from 1975. The 2018 Farm Bill mandated the additional consideration of current food prices that resulted in increased benefits^(7)^.

2 The results from the full model that included all stores were no different in significance or direction from the results presented here.
